# Illuminating microflora: shedding light on the potential of blue light to modulate the cutaneous microbiome

**DOI:** 10.3389/fcimb.2024.1307374

**Published:** 2024-04-10

**Authors:** Hannah J. Serrage, Catherine A. O’ Neill, Natallia E. Uzunbajakava

**Affiliations:** ^1^ Division of Musculoskeletal and Dermatological Sciences, School of Biological Sciences, Faculty of Biology, Medicine and Health, University of Manchester, Manchester, United Kingdom; ^2^ TNO, Holst Centre, Eindhoven, Netherlands

**Keywords:** blue light, microbiome, cutaneous, skin, photobiomodulation, photodisinfection, chromophore, atopic dermatitis

## Abstract

Cutaneous diseases (such as atopic dermatitis, acne, psoriasis, alopecia and chronic wounds) rank as the fourth most prevalent human disease, affecting nearly one-third of the world’s population. Skin diseases contribute to significant non-fatal disability globally, impacting individuals, partners, and society at large. Recent evidence suggests that specific microbes colonising our skin and its appendages are often overrepresented in disease. Therefore, manipulating interactions of the microbiome in a non-invasive and safe way presents an attractive approach for management of skin and hair follicle conditions. Due to its proven anti-microbial and anti-inflammatory effects, blue light (380 – 495nm) has received considerable attention as a possible ‘magic bullet’ for management of skin dysbiosis. As humans, we have evolved under the influence of sun exposure, which comprise a significant portion of blue light. A growing body of evidence indicates that our resident skin microbiome possesses the ability to detect and respond to blue light through expression of chromophores. This can modulate physiological responses, ranging from cytotoxicity to proliferation. In this review we first present evidence of the diverse blue light-sensitive chromophores expressed by members of the skin microbiome. Subsequently, we discuss how blue light may impact the dialog between the host and its skin microbiome in prevalent skin and hair follicle conditions. Finally, we examine the constraints of this non-invasive treatment strategy and outline prospective avenues for further research. Collectively, these findings present a comprehensive body of evidence regarding the potential utility of blue light as a restorative tool for managing prevalent skin conditions. Furthermore, they underscore the critical unmet need for a whole systems approach to comprehend the ramifications of blue light on both host and microbial behaviour.

## Introduction

The integumentary system is home to a diverse and delicately balanced ecosystem, termed the microbiome. It comprises a dynamic community of fungi, bacteria, viruses, archaea and mites, the abundance of which varies considerably across sebaceous (e.g. forehead), moist (e.g. toe webs) and dry (e.g. volar forearm) sites. Collectively, the microbiome participates in a symbiotic dialog with its host, modulating host innate immune responses, maintaining immune homeostasis, protecting against oxidative stress and invading pathogens and, metabolising host products, as well as promoting barrier repair and recovery following insult (Byrd et al., 2018). Tipping this balance can result in dysbiosis, a phenotype often characterised by reduced microbial diversity or overrepresentation of certain species. This dysbiosis is increasingly prevalent in integumentary system conditions, especially in acne vulgaris, atopic dermatitis, seborrheic dermatitis, alopecia areata, psoriasis and non-healing chronic wounds (Kong et al., 2012; Constantinou et al., 2021; Carmona-Cruz et al., 2022; Lawrence et al., 2022). The changes to the microbiome observed range from an elevated abundance of at least *Staphylococcus aureus* in atopic dermatitis to an overrepresentation of specific *Cutibacterium acnes* phylotypes in acne vulgaris (Kong et al., 2012; Lee et al., 2019; Versey et al., 2021).

UV light has anti-microbial action (Marasini et al., 2021), but its carcinogenicity must be considered. Recently, described as a potential ‘magic bullet’ for the 21^st^ century, blue light (400 – 470nm) has proven to be a particularly enticing non-invasive and drug free modality for the management of dysbiosis (Leanse et al., 2022). These antimicrobial effects are often attributed to the expression of porphyrins - heterocyclic compounds with a Soret band at 405nm, by microbes (Serrage et al., 2019). Photo-excitation of porphyrins results in the production of reactive oxygen species (ROS). Optimal dosimetry results in lethal ROS accumulation above a threshold for *Cutibacterium* acnes, a contributor to acne vulgaris (Spittaels et al., 2021).

**Figure 1:**
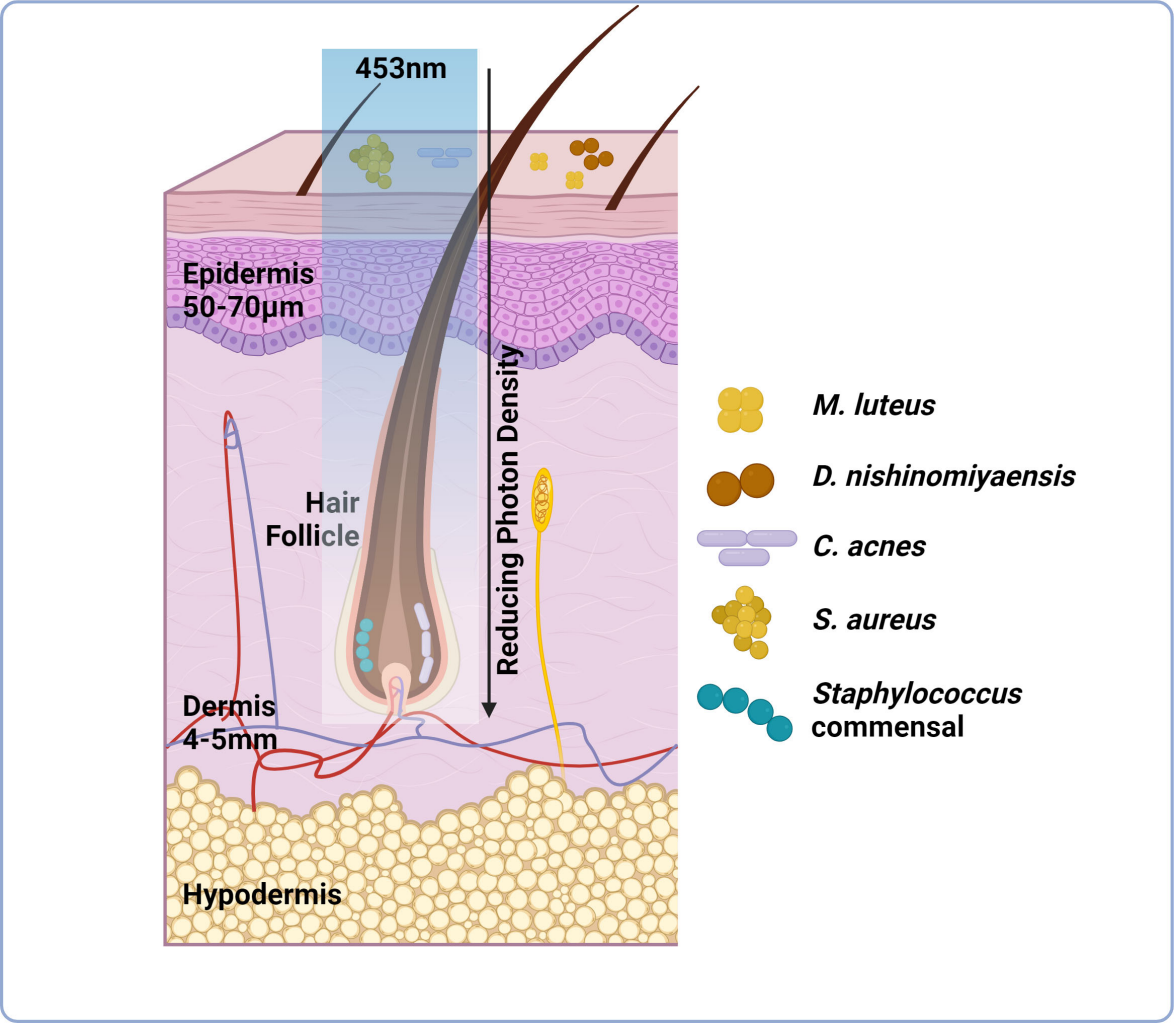
Light transmission through skin is attenuated but can be manipulated through increased irradiance and exposure times. Skin can be subdivided into three layers; epidermis, dermis and hypodermis. Skin microbiome composition is variable these layers Certain species preferentially colonise the skin surface including *M. luteus* and *S. aureus*, whilst others prefer anaerobic environments (hair follicle, dermis) including *P. acnes.* This niche specific colonisation potentially dictates the capacity of light to manipulate skin microbiome composition as well as host responses. Light delivery at 453nm is attenuated through skin by a factor of 2.5-10x at a depth of 0.5-1mm from the skin surface. Light delivery can be improved through increased irradiance and exposure times to a maximum safe exposure of up to 250mW/cm². However, delivery still reduces as the light penetrates through the multilayered structure of skin (as indicated by the reducing photon density observed when passing 453nm light through tissue), therefore diminishing the possibility of manipulating microbial behaviour/viability (image created using BioRender.com).

These findings led to an upsurge of literature suggesting that microbial species respond uniformly to 405nm light, irrespective of the presence or indeed absence of porphyrins. However, such an assumption ignores a myriad of prospective chromophores expressed across the skin and hair follicle microbiome (both in health and disease) that all may exhibit wavelength- and perhaps dose dependent responses to blue light. Indeed, members of the skin microbiome expressing blue light absorbing chromophores are common. Mapping of prospective chromophore’s onto genomes of the top 10 most abundant bacteria of the skin microbiome showcases flavins, porphyrins and carotenoid pigments capable of detecting and responding to the blue component of the visible spectrum (**Table 1**). Expression of such pigments and proteins has been observed from members of the healthy skin microbiome and in species overrepresented in skin conditions including the carotenoid pigment staphyloxanthin (460nm) in *Staphylococcus aureus* (Dong et al., 2019) and the phenazine pyocyanin (380nm) from *Pseudomonas aeruginosa* ( (El-Fouly et al., 2015) **Figure 1**). Wavelength-dependent excitation of these chromophores can lead to a plurality of versatile responses (Harris, 2023) ranging from phototoxicity, to elevated motility, growth and even invasion of eukaryotic cells (Mussi et al., 2010; Halstead et al., 2019).

**Table 1:** Mapping of prospective chromophore’s onto genomes of the top 10 most abundant bacterial members of our skins diverse microbiome.

Species	Prospective Chromophore	Anticipated wavelength range	Evidence of sensitivity to radiation	Gene ID	Ranked Abundance	Citation
*Corynebacterium tuberculostearicum*	Flavin monoxygenase, Beta-carotene monoxygenase	330 – 480nm	Light inducible carotenoid production in some *Corynebacterium* spp.	I6I74_RS00030 I6I74_RS09990	1	(Kolenc and Quinn, 2019)
*Cutibacterium acnes*	Porphyrin	350 – 500nm (peak ~405nm)	Pulsed/high intensity blue light exerts a bactericidal effect on *C. acnes*	PAGK_RS00150	2	(Ashkenazi et al., 2003)
*Staphylococcus epidermidis*	TCA cycle (NADH/FADH)	330 – 465nm	Blue light induces bactericidal effects via a ROS dependent mechanism	EQW00_RS09380	3	(Ramakrishnan et al., 2016; Kolenc and Quinn, 2019)
*Staphylococcus hominis*	Electron Transport chain (NADH/FADH)	330 – 465nm	Evidence of elevated colonisation in atopic dermatitis patients following exposure to UVB	EGX58_RS02040	4	(Kolenc and Quinn, 2019)
*Staphylococcus capitis*	Strain dependent carotenoid production, TCA (NADH/FADH)	330 – 480nm	Evidence of yellow pigmentation in certain strains could confer environmental protection.	NF392_RS09320	5	(Kolenc and Quinn, 2019; Siems et al., 2022)
*Streptococcus mitis*	NADH containing redox sensor	330 – 375nm	Blue light (425nm) exerts no significant effect on biofilm formation at doses up to 192J/cm²	TZ90_RS04770	6	(Ahmed and Claiborne, 1989)
*Micrococcus luteus*	Carotenoid	415 - 480nm	Carotenoid pigment confers protection against UVB	crtX	7	(Mohana et al., 2013)
*Corynebacterium simulans*	Flavin Dependent Oxidoreductase	330 – 465nm	N/A	WM42_RS10275	8	(Kolenc and Quinn, 2019)
*Staphylococcus warneri*	TCA cycle (FADH/NADH)	330 – 465nm	UVC induces phototoxicity, no evidence of the efficacy of blue light	D3P10_RS09700	9	(Shoults and Ashbolt, 2019)
*Streptococcus oralis*	NADH containing redox sensor Porphyrin	330 – 500nm	UVB, but not UVA or blue light (448nm) induce photoxicity up to a maximal dose of 0.6J/cm²	EL140_RS05180 EL140_RS03205	10	(Ahmed and Claiborne, 1989)

Having at hand a ‘magic bullet’ with anti-bacterial action may seem a very efficacious solution. However, as human skin and hair follicle microbiota is a universe of many different species, one needs to restore the ecosystem balance instead of eradicating ‘‘all life’’. This approach brings a challenge of identifying molecular targets for light therapy, suitable wavelength, and treatment regimes.

In this review we first present evidence of the diverse blue light-sensitive chromophores expressed by members of the skin microbiome. Subsequently, we discuss how blue light may impact the dialog between the host and its skin microbiome in prevalent skin and hair follicle conditions. Finally, we examine the constraints of this non-invasive treatment strategy and outline prospective avenues for further research.

## Microbial chromophores

Historically, light sensing systems were thought to be exclusive to photosynthetic organisms However, a growing body of evidence points to a plethora of pigments as well as flavin and porphyrin containing sensing systems in non- photosynthetic bacteria. Here, we summarise these characterised systems and potential implications for future investigations using blue light.

### Flavins

Flavins, including flavin mononucleotide (FMN) and flavin adenine dinucleotide (FAD) can be excited by blue light (Losi and Gärtner, 2011). Herein, we detail two bacterial flavin-containing systems.

#### LOV and blue light-dependent transcription regulation

In 2007 Swartz et al. made the discovery that *Brucella abortis*, a gram-negative bacterium implicated in cutaneous infections in humans (Karaali et al., 2011), expressed a blue light activated, two component system; the light, oxygen or voltage (LOV) histidine kinase. LOV senses blue light via a non-covalently bound flavin co-factor, its photoactivation results in elevated *B. abortis* proliferation and stimulation of macrophage invasion (Kennis and Crosson, 2007; Swartz et al., 2007).

Curiously, LOV domains are encoded in 16% of sequenced bacterial genomes and appear ubiquitous across the *Pseudomonas* genus, with the wound associated pathogen *Pseudomonas aeruginosa* possessing a “short” LOV domain (Glantz et al., 2016). Indeed, blue light was reported effective in photo disinfection of *P. aeruginosa* associated biofilm (Kahl et al., 2022). However, uncertainties persist regarding suitable irradiation parameters.

#### BLUF

Blue-light sensing using flavin (BLUF) domains were first observed ~20 years ago in the single celled algae *Euglena gracilis* and facultative purple photosynthetic bacterium *Rhodobacter sphaeroides* (Park and Tame, 2017). Like the LOV domain, BLUF isn’t exclusive to photosynthetic/phototrophic organisms and it has been observed within the small blue-light-sensing protein A (BlsA). BlsA is expressed by the nosocomially acquired pathogen *Acinetobacter baumannii* (Mussi et al., 2010; Brust et al., 2014). *This* species has proven an increasing problem in hospitals due to its growing recalcitrance to antibiotic treatment, capacity to form biofilm and cause soft-tissue infections (Dijkshoorn et al., 2007).

Upon excitation at 470 nm, BLUF undergoes a conformational change resulting in the binding of flavin adenine dinucleotide (FAD) between two α-helices (Tokonami et al., 2022). In the strain where the BLUF domain was originally observed, *A. baumannii* ATCC 17978, this small protein can drastically impact biofilm formation, virulence and even motility. BLUF domains are observed across a diverse array of microbial species including *Escherichia coli* which is frequently isolated from skin and soft tissue infections (Petkovsek et al., 2009). *E. coli* possesses a BLUF-EAL protein (YcgF) which binds the MerR-like repressor (YcgE) upon illumination, resulting in dissociation from its operator DNA and regulation of two component systems that diminish biofilm formation and adhesion to surfaces (Tschowri et al., 2009). Details of BLUF diversity, structure and function are discussed in detail elsewhere (Kaushik et al., 2019).

### Porphyrins

Porphyrins are a highly abundant group of macromolecules, implicated in the biosynthesis of heme and chlorophyll (Bonkovsky et al., 2013). This group of heterocyclic organic compounds act as pigments, exhibiting a Soret band at 400 - 420nm (Gouterman, 1978; Koren et al., 2011). Excitation of this Soret band results in ROS accumulation and ultimately cell lysis (Wu et al., 2018). Biosynthesis of porphyrins includes generation of δ-aminolevulinic acid from glutamyl-tRNA (Jones et al., 2020), ultimately resulting in downstream accumulation of protoporphyrin IX, uroporphyrin III and coproporphyrin III (Shu et al., 2013).

The abundant skin commensal *Cutibacterium acnes* synthesises a range of porphyrins (Johnson et al., 2016). This has been exploited for utilizing blue light for management of the *C. acnes* in acne vulgaris (Sorbellini et al., 2018). However, success of this treatment depends upon a host of factors including the propensity of *C. acnes* strains to produce porphyrins. The latter highly varies and depends upon the presence of the porphyrin biosynthesis repressor gene; *deoR* (Spittaels et al., 2021). This may explain subsets of acne patients proving recalcitrant to blue light treatment (Diogo et al., 2021). However, other factors including abnormalities in follicular keratinisation and sebum overproduction contribute to onset (Cruz et al., 2023). Therefore, failing to address other underlying causes may also contribute to the persistence of this prevalent condition.

Intriguingly, the expression of porphyrins occurs across a host of wound-associated pathogens. Jones et al. surveyed the porphyrin producing and autofluorescence of 32 bacterial members of the wound microbiome and found 88% exhibited porphyrin expression and excitation with blue light (Jones et al., 2020). This suggests that violet-blue light could be a promising approach for infection management.

### Pigments

Pigments are expressed by a diverse range of both environmental and skin-resident microbes. Expression of such pigments is hypothesised to confer potential selective advantages including both photo- and thermal- stability (Celedón and Díaz, 2021; Agarwal et al., 2023). However, one still needs to comprehend how we might exploit these traits to manage microbial adherence and biofilm formation in both health and disease scenarios (Narsing Rao et al., 2017).

#### Yellow pigmented photoreceptors

Bacteria displaying yellow pigmentation are common across the skin microbiome. Commensals including *Micrococcus luteus* (Anwar and Prebble, 1977), *Kocuria rizophila* (Govindan et al., 2020), *Dermacoccus nishinomiyaensis* (Klein et al., 2017) and *Staphylococcus aureus* (Zhang et al., 2018) exhibit a golden yellow colour which is derived from a carotenoid pigment, a molecule with a profound antioxidant function. Its absorption displays peaks across 415 – 485nm, indicating photosensitivity to blue light (Darvin et al., 2022). Similarly, group B streptococci (*Streptococcus agalactiae*, gastrointestinal tract commensal often associated with skin and soft tissue infections including diabetic foot ulcers (Raabe and Shane, 2019),) express granadaene, a yellow/orange pigment displaying an absorption spectra from 435-485nm (Rosa-Fraile et al., 2014).

#### Phenazines

Phenazines form part of a group of nitrogen containing heterocyclic compounds (Pierson and Pierson, 2010). They shuttle electrons, ultimately modifying cellular redox status thereby regulating gene expression, biofilm formation and even architecture (Wang et al., 2011; Jo et al., 2020). The colour of this metabolite varies from yellow to blue, depending upon pH or redox state with absorption band ~385 – 500nm across the UVA-green range (Ryazanova et al., 2007).

One well-characterised derivative of a phenazine is pyocyanin, a blue metabolite expressed by the pathogen *Pseudomonas aeruginosa*, proven to facilitate aggregation via extracellular DNA (Das et al., 2013), promote biofilm formation and maintain bacterial fitness (Jayaseelan et al., 2014). The absorption spectrum of pyocyanin is dependent upon pH and redox state, with a maximum occurring in the UVA range (~380nm) (El-Fouly et al., 2015). However, purified pyocyanin can potentiate the bactericidal properties of 405nm wavelength against methicillin resistant *S. aureus*, indicating its capacity to elicit a response to blue light (Leanse et al., 2022).

## Blue light and cutaneous conditions

Dysbiosis in skin and hair follicle conditions is common, demanding effective strategies to address microbiome composition imbalances. Here, we highlight how blue light may manipulate microbial behaviour in highly prevalent skin and hair conditions, where there remains a deficit in treatments that effectively restore skin health. Moreover, we explore how light exposure may exert dual effects through photoexcitation of host derived chromophores.

### Acne

Acne is a highly prevalent dermatosis affecting up to 95% of 11-30 year olds, characterised by an elevated abundance of *Cutibacterium* spp residing upon affected sites (Golchai et al., 2010). Blue light therapy is a well-established drug-free strategy for management of acne vulgaris (Gold, 2007). The efficacy of blue light treatment is thought to centre on photoexcitation of porphyrin containing *Cutibacterium* spp, resulting in bacterial lysis and resolution of acne vulgaris. Indeed, the bactericidal effects of blue light on *Cutibacterium acnes* have been widely reported (Masson-Meyers et al., 2020; Nakayama et al., 2023), and porphyrin containing phylotypes appear more abundant in acne vulgaris (Huang et al., 2023).

Advances in a metagenomic sequencing have revealed that next to *C. acnes*, as the key player in acne pathology, other species may play an active role in promoting disease pathogenesis. The abundance of *Staphylococcus* spp. increases with severity of acne (Dreno et al., 2017). In particular, *S. epidermidis* has been cited as a new player in acne pathogenesis. *Staphylococcus epidermidis* is abundant in acne lesions and utilises glycerol as a shared carbon source, resulting in production of short chain fatty acids (SCFA) (Wang et al., 2014). These SCFAs facilitate competition between *S. epidermidis* and *C. acnes*, indicating a delicate balance between these two species could be important in the resolution of inflammation associated with acne vulgaris. *S. epidermidis* possesses a NADH:flavinoxidoreductase, which may be excited by blue light (Wu et al., 2015) and *Ramarkrishnan et al.* indicated exposure to 405nm light resulted in cell cytotoxicity via a ROS dependent mechanism (Ramakrishnan et al., 2016). However, studies also indicate *S. epidermidis* possesses mechanisms by which it can deactivate ROS (Balasubramaniam et al., 2020; Smith et al., 2023). Understanding of how these protective responses may defend against blue light exposure and modulate imbalances in microbiota composition remains to be elucidated.

### Psoriasis

Psoriasis is a long-term, chronic immune-mediated skin disease with no cure. It is linked to comorbidities like psoriatic arthritis, cardiometabolic issues, and mental health conditions with a prevalence of 3% in adults in the USA (Armstrong et al., 2021). In the 1990s, researchers discovered psoriatic plaques in 45% of patients emitted red fluorescence when excited with blue (442nm) light (Bissonnette et al., 1998). Autofluorescence was attributed to protoporphyrin IX, elevated in this subset of patients (Bissonnette et al., 1998). Auto-fluorescence positive lesions have proven to correlate with psoriasis severity and identified as a novel approach for psoriasis diagnosis (Wang et al., 2017). It was hypothesised that, like with acne vulgaris, protoporphyrin IX may be of microbial origin, indicating inter-individual differences in skin microbiome may predispose towards porphyrin accumulation. Presently, literature citing psoriasis community composition remains variable (Mazur et al., 2021) and no discernible link between microbiome composition and protoporphyrin accumulation was identified. Yet, a growing body of literature supports the use of blue light for treating psoriasis, where efficacy varies from improved disease severity (Lesiak et al., 2021) to no significant effect, specifically in a subset of patients with elevated endogenous protoporphyrin’s (Maari and Bissonnette, 2002). Favourable patient outcomes were attributed to antiproliferative and pro-differentiative effect of blue light on keratinocytes shown *in vitro* and to nitric oxide-mediated mechanism shown *in vivo*, overall resulting in resolution of plaques (Sadowska et al., 2021).

Perturbations in the microbiome associated with psoriasis also include enrichment in members of the *Pseudomonas* genus, particularly sensitive to blue light exposure (Chen et al., 2020). Future studies shall characterise the cross-talk between the microbiome and skin in this multi-faceted and highly prevalent condition.

### Atopic dermatitis

Atopic dermatitis (AD) is one of the most common chronic, relapsing inflammatory skin disorders affecting up to 3% of adults worldwide (Kapur et al., 2018). It is characterised by an itch-flare cycle that can lead to lichenification (hyperpigmentation, skin thickening and exaggerated skin lines) AD pathogenesis is characterized by impaired epidermal barrier function and immune dysregulation some of which may be driven by *S. aureus*, which is over-abundant in the skin of patients with AD (Weidinger et al., 2018).. Current treatments both topical (tacrolimus, corticosteroids and antibiotics) and systemic (cyclosporine) act to supress immune responses and diminish *S. aureus* abundance (Belloni Fortina and Neri, 2015; Avena-Woods, 2017). However, these first line treatments are often ineffective and there remain cohorts of patients recalcitrant to mainstay treatments for AD (Lewis and Feldman, 2018).

A growing body of literature has endeavoured to evaluate the efficacy of visible – near infra-red (NIR) light in the management of AD. Blue light has garnered considerable attention due to its capacity to manipulate dendritic cell activation, resulting in modulation of cell proliferation and inflammatory responses *in vitro* (Liebmann et al., 2010; Fischer et al., 2013). *In vivo* studies demonstrated that full body irradiation with blue light (400 - 500 nm) resulted in improved pruritus (urge to itch), quality of life and reduced hydrocortisone use (Uzunbajakava et al., 2023).

It is yet to be understood how blue light manipulates the AD microbiota. *Staphylococcus aureus* is enriched in AD flares (lesional skin) (Kong et al., 2012) and is capable of manipulating dendritic cell activation and pruritus via induction of IL-31, ultimately promoting skin barrier dysfunction (Wu and Xu, 2014; Blicharz et al., 2019). *Staphylococcus aureus* exhibits a golden yellow colour due to the expression of carotenoid pigment; staphyloxanthin, which displays characteristic absorption peaks at 408nm and 486nm (Harris, 2023). The expression of this pigment promotes bacterial survival, where in environments with high levels of oxidative stress staphyloxanthin acts as an antioxidant, detoxifying free radicals facilitating enhanced survival both *in vitro* (Clauditz et al., 2006) and in murine models for chronic wounds (Campbell et al., 2023), an environment associated with high levels of oxidative stress (Cano Sanchez et al., 2018).

This potential carotenoid-dependent tolerance to radiation is not exclusive to *S. aureus*. For example, skin commensals *M. luteus*, *Kocuria* spp. and *D. nishinomiyaensis* exhibit carotenoid pigment expression (Koshti et al., 2022). *M. luteus*, a skin commensal with probiotic properties, has proven highly tolerant to UV exposure due to carotenoid expression but responses to blue light are yet to be evaluated (Anwar and Prebble, 1977). Future steps will be required to understand how blue light manipulates pigment producing bacteria and in turn, how we might exploit these characteristics for management of prevalent conditions including AD.

### Alopecia areata

Alopecia areata (AA) is an autoimmune disease with a lifetime incidence of 2.1% characterised by the collapse of immune privilege in the hair follicle which causes hair loss and has drastic impacts on patient quality of life, often causing psychological distress (Pratt et al., 2017).

Since early discovery of positive effect of red light on hair regrowth (Hamblin, 2016), strides have been taken to understand whether red light could prove a strategy for management of alopecia areata as well as to unravel the fundamental mechanisms behind photobiomodulation of the hair cycle. Blue light has been reported to prolong anagen hair growth phase via sustained proliferation in the hair follicle matrix (Buscone et al., 2017). The effect has been attributed to interaction of blue light with putative photoreceptors, cryptochromes and opsins (Buscone et al., 2017; Buscone et al., 2021). However, so far to the best or our knowledge no research has been conducted to unravel a potential blue-light-dependent hair growth modulation via modulation of the HF microbiome (Lodi et al., 2021).

The collapse of immune privilege is considered a pathogenic event in AA and patients exhibit an elevated abundance of *Cutibacterium* spp. and *Mallasezia* spp, and reduced abundance of *Staphylococcus* spp (Huang et al., 2019; Sánchez-Pellicer et al., 2022). Increasingly, researchers are posing the question of whether the scalp microbiome can influence hair regrowth. The antibiotic roxithromycin elevates hair growth, but is also toxic to *C. acnes* (Sadowska et al., 2021). The fungicidal and bactericidal effects of blue light on both *Cutibacterium* and *Mallasezia* spp. have been reported (Wi et al., 2012; Nakayama et al., 2023). Therefore, understanding of how blue light manipulates microbiome communities and markers for AA pathogenesis warrants investigation.

### Chronic wounds

Prevalence of chronic nonhealing wounds represent an immense and ever-growing global pandemic, with incidence and mortality rates exceeding those of many common cancers (Weigelt et al., 2022). Recently, researchers started exploiting light to understand wound microbiome composition. Excitation using a violet-blue light (~405nm) resulted in detection of auto-fluorescence in the orange, red and green spectral regions, denoting the presence of *S. aureus, A. baumannii* and *P. aeruginosa*, respectively (DaCosta et al., 2015; Jung et al., 2022; Rahma et al., 2022). This auto-fluorescence has been observed in >50% of patients, and is increasingly utilised to detect infection. Violet-blue light can be absorbed by several microbiome species and could be potentially applied for photo-disinfection (Bayat et al., 2022).

Studies assessing blue light mediated photodisinfection addressed readily isolated wound pathogens; *P. aeruginosa* and *S. aureus*. Mechanisms of blue light dependent manipulation of *P. aeruginosa* viability and biofilm formation have been assessed in single species biofilm models, reporting blue light-dependent manipulation of membrane potentials in *P. aeruginosa*, resulting in altered biofilm formation (Kahl et al., 2022). Blue light induction of membrane depolarisation/potential has proven both dose and biofilm formation stage dependent: *P. aeruginosa* exhibits heightened sensitivity to blue light in the adherence (1h) and proliferation (3h) stages of biofilm formation. Cells in the proliferative stage are particularly sensitive and doses <20mJ/cm² have been exploited to disperse biofilm (Blee et al., 2020). This poses the question; could blue light be applied as a preventative measure following injury to prevent colonisation by *P. aeruginosa* alongside treatment of chronic wounds?

Clinical studies on blue light efficacy in non-healing wounds are scars, with only one study using blue light to accelerate wound healing (Fraccalvieri et al., 2022). Therefore, future studies should endeavour to evaluate promising bactericidal and wound healing properties of blue light (Bayat et al., 2022).

## The future of blue light in skin health: potential therapeutic insights and challenges

Exposure of skin to blue light has become a contentious topic, sparking discussions over its benefits and risks, implications in disease management, circadian clock, pigmentation but also photodamage (Campbell et al., 2017; Stern et al., 2018). Blue light could be a promising therapy for a diverse range of cutaneous and systemic conditions, from blood pressure reduction to management of seasonal affective disorder (Meesters et al., 2016; Stern et al., 2018).

However, reports of adverse effects have also proven abundant, even from exposure to environmental levels of blue part of electromagnetic spectrum (Diogo et al., 2021; Kumari et al., 2023). These potentially undesired effects span erythema (sunburn), photoaging, decreased skin hydration, elevated melanin production and subsequent hyperpigmentation (Mahmoud et al., 2010; Duteil et al., 2014; Duteil et al., 2020; Kumari et al., 2023). However, the latter has also been hypothesized to carry a photoprotective effect against subsequent UV irradiation by some groups (Uzunbajakava et al., 2023). The exposure to blue light has only increased as technological advances have resulted in our constant exposure to blue light from screens and mobile devices. The actual irradiance and dose emitted by these appliances is far lower than those found in natural lighting and hence the impact of such appliances on the skin is minimal, if present at all (Arjmandi et al., 2018; Duteil et al., 2020; Kumari et al., 2023; Uzunbajakava et al., 2023). Nevertheless, exposure to such devices, peaking around ~440nm via visual pathways, has been shown to manipulate circadian rhythm through excitation of melanopsin located within retinal ganglion cells (Wahl et al., 2019). Despite increased understanding of the dose dependent effects of blue light on skin, there remains limited literature exploring how blue light might manipulate the healthy skin microbiome. In one study, Willmott et al. evidenced sunlight exposure dependent shifts in microbiome composition in holidaymakers. It is hypothesised blue light could play an important role in mediating these shifts, due to the application of sunscreens by holiday makers that may mitigate prospective effects of other forms of radiation (UVA/UVB (Willmott et al., 2023)).

Notably, as humans, we have evolved under the sun, and under the influence of the blue spectral band, where the skin is directly exposed to photons of light. The intensity of the blue band varies considerably depending upon the time of day (Bird and Riordan, 1984). It’s highest intensity relative to other spectral components occurs at noon, but reduces significantly at dusk. Hence it is not surprising that blue light entrains a circadian rhythm that is controlled via central control mechanism (CNS) but perhaps also locally via our cutaneous system of clock genes (Lyons et al., 2019). These circadian rhythms are intrinsically linked to skin diseases. For example, symptoms of psoriasis exhibit a diurnal pattern with a more severe itch in the evening for 70% of patients (Duan et al., 2021). Circadian oscillations also affect mast cells (Baumann et al., 2013), participating in various immune and inflammatory reactions, including allergies, infections, wound healing and skin cancer. In line with that, patients with mastocytosis present higher plasma histamine levels in the morning (Friedman et al., 1989) and patients with chronic urticaria often have symptoms in the evening (Maurer et al., 2009).

*In vivo* studies have revealed deletion of *Clock* genes in mouse models for atopic dermatitis result in more severe delayed-type allergic reactions and elevated ‘nocturnal pruritus’ in patients (Lavery et al., 2016; Duan et al., 2021). These diurnal cycles are certainty not restricted to the skin cells only, as the skin together with its microbiome evolved under the sunlight. So, it is not surprising that the relative abundance of members of the skin microbiome vary with the time of day. For example, evidence suggests that the abundance of acne associated species *C. acnes* increases in the evening (Wilkins et al., 2021). This highlights a key question surrounding blue light therapy: could efficacy depend upon the time of exposure?

Perhaps the most promising trait of blue light is its potential to address dysbiosis in skin and hair follicle conditions, while exerting little or no impact on bacterial resistance following repeated exposures (Haridas and Atreya, 2022). The rising prevalence of skin diseases urgently demands a comprehensive understanding of the dialog between the host and microbiome in response to blue light to develop adequate therapies for relevant conditions. Future studies are needed as current focus remains upon nosocomial acquired pathogenic species only, with little regard for the contribution of commensals to light dependent responses. For example, abundant members of the commensal microbiome express carotenoid pigments, that exhibit antioxidant properties (Mohana et al., 2013). It could be hypothesised that strains may evolve to elevate expression of carotenoid synthesis genes as a protective mechanism against blue light exposure. Another aspect to consider is virulence, where reports cite blue light enhanced virulence of *S. aureus* and *A. baumannii* (Tuttobene et al., 2021).

**Figure 2:**
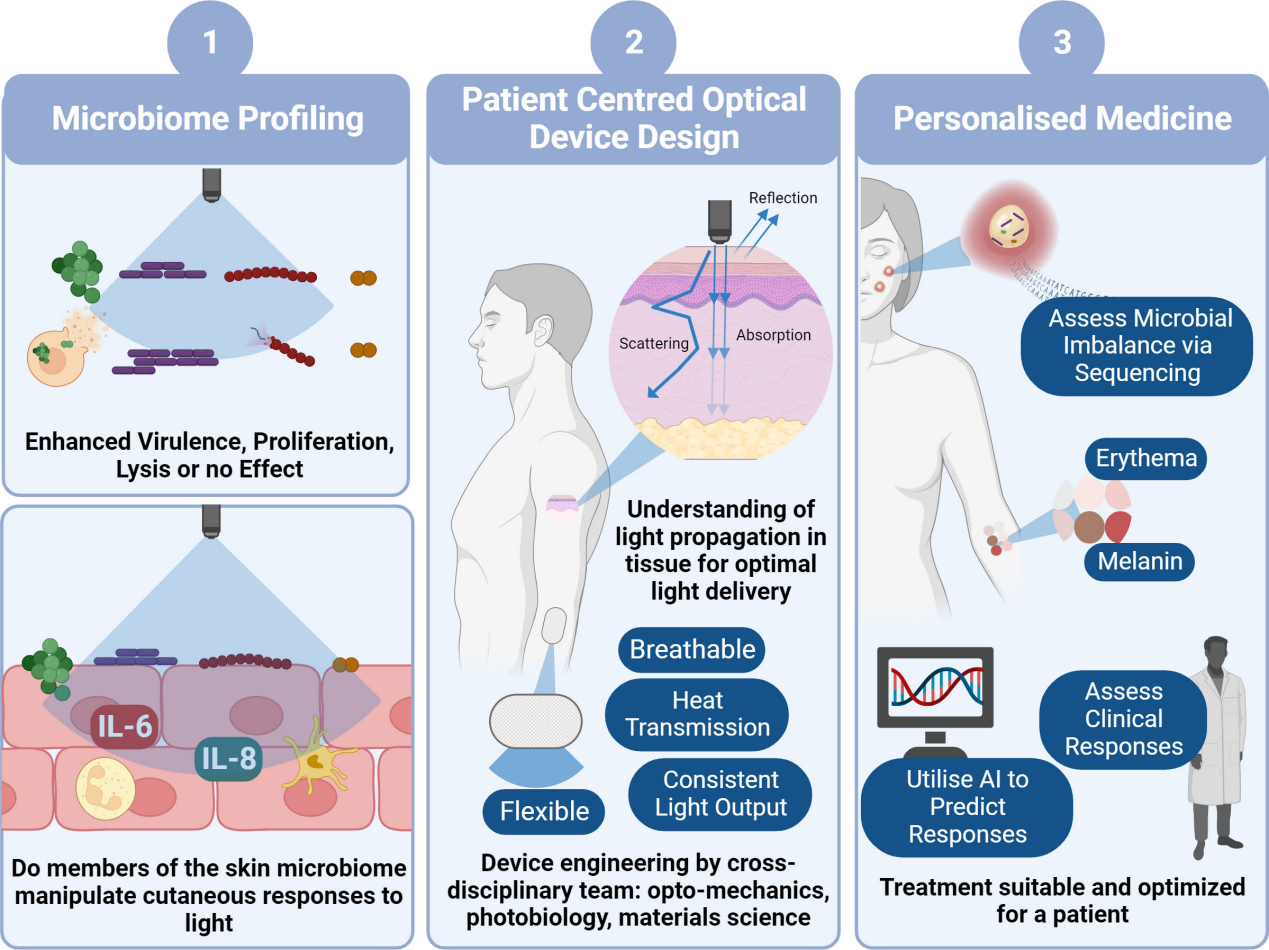
Pathway to Clinical Implementation of Blue Light for Management of Skin Conditions. To determine the viability of blue light as a potential treatment option for skin conditions, firstly we need to decipher how blue light impacts microbial behaviour and in turn how theses microbes manipulate host responses to blue light utilising laboratory characterisation techniques (1). This will involve development of increasingly physiologically relevant models with the eventual use of ex vivo models and microbes derived directly from individuals. In parallel with this we also must ensure device design (2) is central to clinical application through thorough characterisation of light transmission through skin and patient centred design of photonics devices. This will involve a combination of photonics experts, biologists and engineers to reliably characterise a device convenient for everyday use. Ultimately, design of a device and application of irradiation of parameters will also rely upon personalised approach to medicine (3). For example, determining if there is a microbial imbalance in tissue and in turn anticipating whether the microbiome will elicit a favourable response to blue light, without elevating virulence and promoting host cell tissue apoptosis. We all must consider the dose dependent response where some individuals may experience a ‘sunburn’ response to blue light, whilst others may experience hyperpigmentation through elevated melanin expression. Such responses may be predicted through clinical consultation or perhaps development of AI platforms that automatically predict responses. Collectively this pathway will facilitate prediction of responses and suitability for clinical applications (image created using BioRender.com).

To achieve favourable patient outcomes, effective blue light delivery systems will be required. Blue light is strongly attenuated by the skin haemoglobin and melanin in comparison to wavelengths within the red and near-infrared spectra. Still blue light can reach relatively deep targets in the skin, including the hair follicle bulge at a depth of approximately 0.5-1mm relative to the skin surface, while its intensity will be highly attenuated versus the photon density administered at the skin surface (**Figure 2**) (Uzunbajakava et al., 2023). While blue light may be most effective in manipulating surface level microbes, its efficacy in influencing sub-epidermal layers including sebaceous gland and entire hair follicles will be modulated by light attenuation by the skin. Methods to boost photon concentration at deeper-located targets include usage of a larger spot size, pulsing along with increasing the applied dose within the safety limits, and perhaps usage of light guides along the hair follicle shafts for light delivery to a deeper targets (Ash et al., 2017). Therefore, critically the relationship between the location of the target, penetration depth, dose, irradiance and pulsing in relation with efficacy and potential adverse effects must be considered when designing blue light devices.

**Figure 3:**
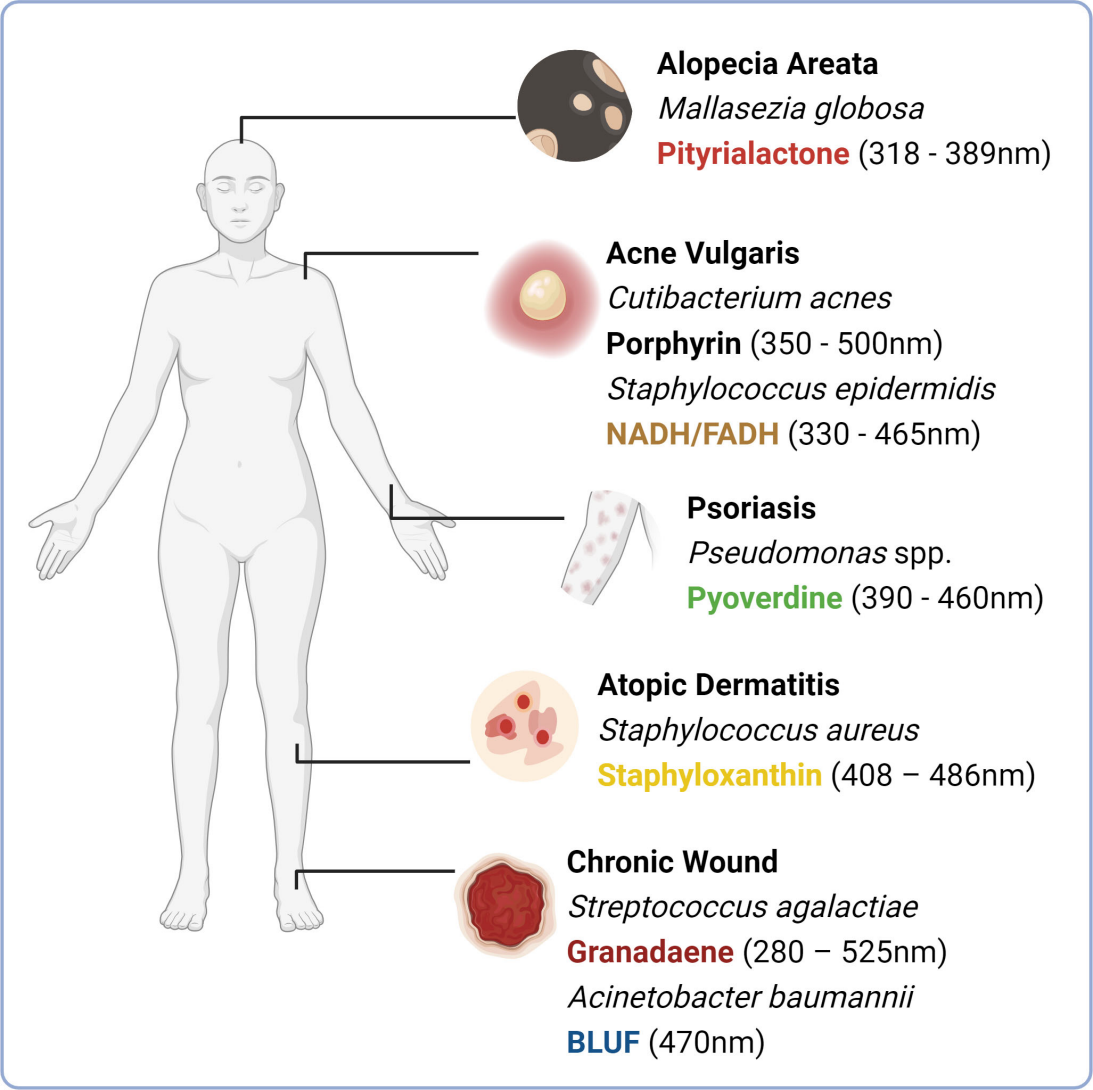
Chromophores expressed by microbial species implicated in skin and hair conditions. An imbalance in skin microbiome composition is often observed in skin and hair follicle conditions including alopecia areata (elevated abundance of Mallasezia globosa), acne vulgaris (increased abundance of porphyrin producing phylotypes of Cutibacterum acnes), psoriasis (possible association with the elevated abundance of Pseudomonas aeruginosa) and atopic dermatitis (increased abundance of Staphylococcus aureus in atopic dermatitis flares). Soft tissue infections that can ultimately develop into chronic wounds are often colonised by environmentally acquired isolates including Streptococcus agalactiae and Acinetobacter baumannii, which express blue light absorbing chromophores granadaene and blue light sensing using flavin (BLUF) respectively. This figure depicts these species commonly overrepresented, alongside their prospective chromophores. (Image created with Biorender.com).

It is apparent that an interdisciplinary and whole systems approach is required for the effective translation of blue light to clinics (**Figure 3**). However, alongside understanding individual microbial responses to blue light, development of co-culture models to assess the impact of microbes on host-responses and an understanding of light transmission through tissue, the patient must be at the centre of light delivery. Device design must be carefully considered, and recent technological advances have facilitated the development of flexible, breathable, and convenient devices capable of effective light delivery (Matsuhisa et al., 2021). Such designs should be incorporated into a personalised approach to blue light exposure. In which a combination of microbiome composition, skin condition severity and survey of pre-existing conditions (e.g., cancer) should be taken into consideration prior to exposure.

## Conclusion

Blue light represents an attractive non-invasive, drug-free approach for management of cutaneous conditions where there is an imbalance in microbial community composition. However, we have only scratched the surface in understanding the relative abundance of blue light sensing systems across the skin microbiome, and in turn how this impacts microbial viability, motility and virulence. It is apparent that steps must be taken in this nascent field to understand how presence or absence of these systems manipulates microbial community composition and in turn how photosensitive chromophores expressed by the host contribute to these responses.

## Data availability statement

The raw data supporting the conclusions of this article will be made available by the authors, without undue reservation.

## Author contributions

HS: Conceptualization, Investigation, Methodology, Project administration, Writing – original draft, Writing – review & editing. CO’N: Writing – review & editing. NU: Writing – review & editing.
